# Increased pathogen exposure of a marine apex predator over three decades

**DOI:** 10.1371/journal.pone.0310973

**Published:** 2024-10-23

**Authors:** Karyn D. Rode, Caroline Van Hemert, Ryan R. Wilson, Susannah P. Woodruff, Kristy Pabilonia, Lora Ballweber, Oliver Kwok, Jitender P. Dubey

**Affiliations:** 1 U.S. Geological Survey, Alaska Science Center, Anchorage, Alaska, United States of America; 2 U.S. Fish and Wildlife Service, Marine Mammals Management, Anchorage, Alaska, United States of America; 3 Colorado State University Veterinary Diagnostic Laboratory, Fort Collins, Colorado, United States of America; 4 US Department of Agriculture, Agricultural Research Service, Beltsville, Maryland, United States of America; Animal Health Centre, CANADA

## Abstract

Environmental changes associated with global warming create new opportunities for pathogen and parasite transmission in Arctic wildlife. As an apex predator ranging over large, remote areas, changes in pathogens and parasites in polar bears are a useful indicator of changing transmission dynamics in Arctic ecosystems. We examined prevalence and risk factors associated with exposure to parasites and viral and bacterial pathogens in Chukchi Sea polar bears. Serum antibodies to six pathogens were detected and prevalence increased between 1987–1994 and 2008–2017 for five: *Toxoplasma gondii*, *Neospora caninum*, *Francisella tularensis*, *Brucella abortus/suis*, and canine distemper virus. Although bears have increased summer land use, this behavior was not associated with increased exposure. Higher prevalence of *F*. *tularensis*, *Coxiella burnetii*, and *B*. *abortus/suis* antibodies in females compared to males, however, could be associated with terrestrial denning. Exposure was related to diet for several pathogens indicating increased exposure in the food web. Elevated white blood cell counts suggest a possible immune response to some pathogens. Given that polar bears face multiple stressors in association with climate change and are a subsistence food, further work is warranted to screen for signs of disease.

## Introduction

Climate warming has contributed to changes in wildlife and zoonotic disease exposure worldwide [1–3]. Shifting ecological conditions can influence the susceptibility of hosts to disease, alter rates of transmission, and facilitate invasion of novel pathogens and parasites [1, 4]. The impacts of disease on animal health are broad-reaching, and include direct morbidity and mortality, reproductive failure, and energetic costs through heightened immune response. In some cases, severe population declines have followed disease outbreaks among free-ranging wildlife [5, 6]. Spillover of zoonotic diseases can also have important consequences for human health, particularly in regions where people and wildlife come into frequent contact [4, 7].

In the Arctic, where warming is occurring at nearly four times the global rate, infectious diseases present a growing concern to both wildlife managers and human communities [8, 9]. Environmental factors, such as temperature, precipitation, and hydrology, can affect parasite and pathogen life cycles, while concomitant shifts in animal movements and migratory patterns create new opportunities for pathogen exchange [7, 10, 11]. For instance, increased use of terrestrial habitats by marine-dominant species such as polar bear (*Ursus maritimus*) and Pacific walrus (*Odobenus rosmarus divergens*) may promote contact between species that were historically segregated [12–14]. Similarly, northward range shifts in some migratory birds [15] and mammals [16, 17] have the potential to change community dynamics and therefore disease ecology [4, 7, 18]. Wildlife often serve as reservoirs and contribute to the movement of pathogens over large distances through migration [8, 19]. Growing demands for resource development and infrastructure in the Arctic and the potential for new overland and marine transport corridors [20, 21] may also affect exposure of wildlife to pathogens of anthropogenic origin via runoff into marine or freshwater habitats, contact with domestic animals, and introduction of invasive species [19, 22]. Pathogens in wildlife can affect human health, either as a direct source of zoonoses or via secondary effects on food security and cultural traditions if animal populations are impacted [19]. However, such risks are difficult to quantify without sufficient knowledge about host diversity or disease prevalence.

Baseline data on the prevalence of pathogen exposure are limited for many Arctic regions and potential impacts on wildlife populations have not been well characterized. With the exception of specific morbidity or mortality events that generate targeted research and diagnostic efforts, pathogen surveillance has generally been opportunistic and, limited to a single point in time. Long-term wildlife disease studies are scarce across the circumpolar Arctic, but where they have been conducted increasing trends in prevalence have been noted, often in association with climate-related factors [10, 23, 24]. In addition to tracking temporal changes, it is important to identify specific host and ecological factors associated with pathogen exposure to better understand how population demographics could be impacted by disease.

Polar bears are a species of conservation concern because of substantial declines in their Arctic Sea ice habitat. They have specialized diets composed primarily of ice-associated seals [25–27] that make them vulnerable to environmental change but also ecological sentinels due to their high trophic level position. In addition, they are important cultural and subsistence resources of Indigenous people in many parts of their range [28]. Determining prevalence, risk factors, and potential impacts of pathogen exposure have been identified as important aspects of monitoring polar bear health [29–31]. Further, as apex predators with the largest home ranges of any carnivore and a circumpolar distribution, tracking disease exposure across polar bear populations is a potentially useful indicator of the presence and prevalence of pathogens within the remote Arctic ecosystems they inhabit.

In the Chukchi Sea, polar bears have experienced some of the highest rates of summer sea ice loss anywhere in the Arctic, with corresponding increases in summer land use [32, 33]. The proportion of bears in the population summering on land increased from 10 to 50% and duration onshore increased from 30 to 60 days between the 1980s and 2000s [32, 33]. Bears that come onshore are often attracted to human garbage and food which has been shown to increase polar bear exposure to pathogens, particularly those of anthropogenic or terrestrial origin [i.e., *Toxoplasma gondii*; 23] and result in a heightened immune response [34]. Compared to other polar bear populations, Chukchi Sea bears have a more southerly distribution, making them potentially susceptible to pathogens that expand northward with warming ocean conditions. Increased pathogen exposure has been reported in polar bears in western Hudson Bay, which is also at the southern edge of the polar bear’s range [24].

**Fig 1:**
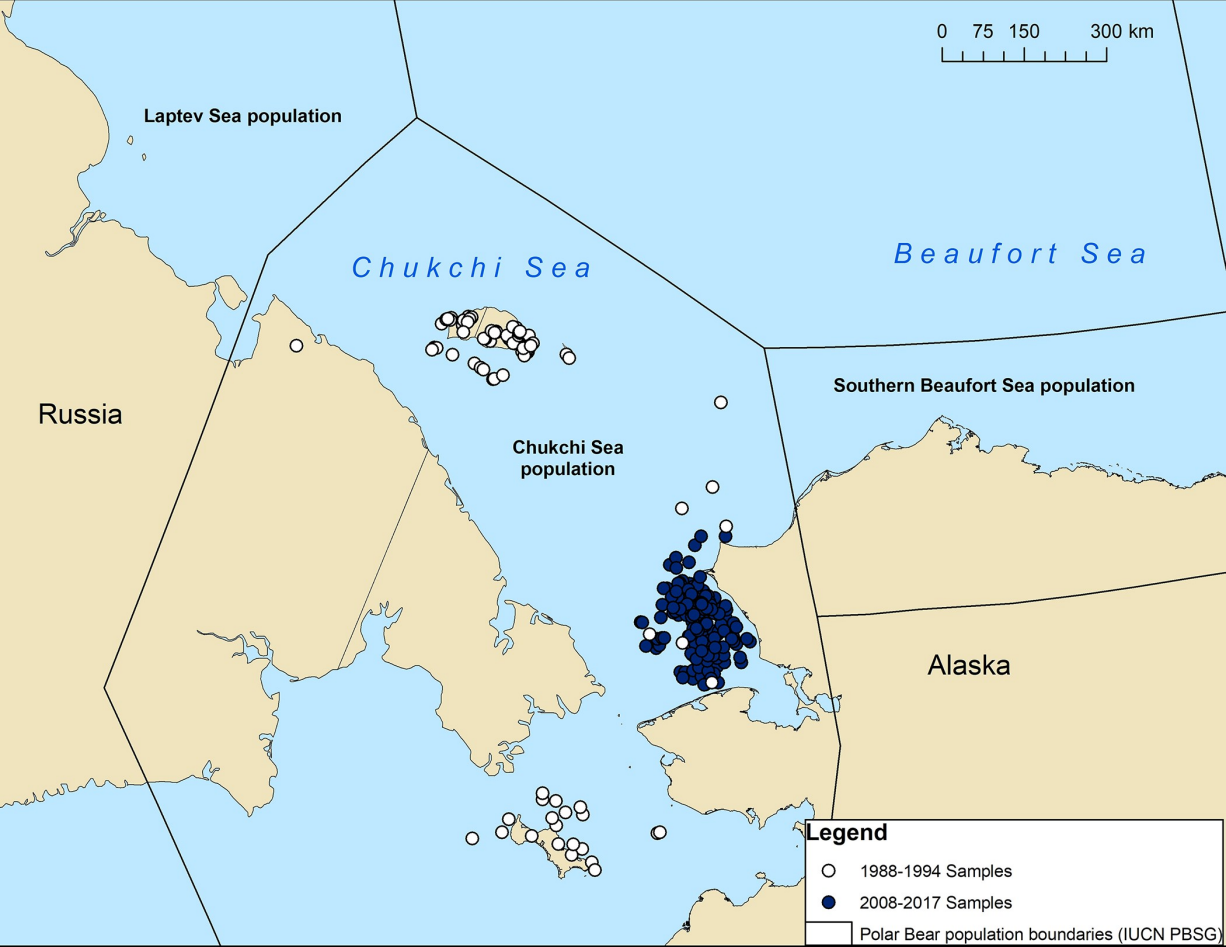
Locations of Chukchi Sea polar bears sampled during two time periods: 1987–1994 and 2008–2017. Population boundaries are based on the International Union for the Conservation of Nature (IUCN) Polar Bear Specialist Group (PBSG) designations.

In this study, we tested for a suite of wildlife and zoonotic pathogens in Chukchi Sea polar bears from available fecal and serum samples collected during ongoing studies in 1987–1994 and 2008–2017 (Fig 1). Our objectives were to 1) evaluate whether pathogen exposure of polar bears in the Chukchi Sea population changed over time, 2) identify factors that might affect individual exposure, including diet, sex, age, maternal transfer, and summer land use, and 3) determine whether exposure might elicit an immune response. We conducted antibody serological tests for *Toxoplasma gondii*, *Neospora caninum*, *Francisella tularensis*, *Coxiella burnetii*, *Leptospira* spp., *Brucella canis*, *B*. *abortus/suis* and canine distemper virus (CDV) and screened fecal samples for *Giardia* cysts and *Cryptosporidium* oocysts. Although multiple species of *Brucella* occur in marine mammals [35, 36], we selected standard *Brucella* tests targeting smooth biovariants (*B*. *abortus/suis*), based on prior findings in polar bears [23, 37, 38], and *B*. *canis*, which is occasionally detected in northern canids [39]. We selected pathogens for inclusion on the basis of prior detection in polar bears, allowing for comparisons across populations and regions; existence of plausible marine and/or terrestrial transmission pathways; and potential relevance to polar bear health. All of these pathogens can cause clinical disease in domestic and wild animals and some have been linked directly to morbidity and mortality in Arctic wildlife [40–43]. For example, morbilliviruses like CDV are known to cause respiratory disease in foxes, seals, and bears [44, 45], with CDV-induced neonatal mortality documented in a polar bear [46]. Clinical toxoplasmosis, which can lead to outcomes including abortion and encephalitis, has been reported in many marine mammal species, such as beluga whales (*Delphinapterus leucas*), sea otters (*Enhydra lutris*), and northern fur seals (*Callorhinus ursinus*) [40, 42, 47]. Most of the pathogens we investigated have also been detected in polar bears or their prey species (e.g., ringed seals [*Phoca hispida*] and bearded seals [*Erignathus barbatus*]) [35, 48, 49], including prey populations in the Chukchi Sea [50–52], thus making them good candidates for surveillance. Additionally, by including several pathogens considered to be primarily of terrestrial origin, we further explored the possibility that land-based or nearshore exposure may be important; these include *N*. *caninum*, *F*. *tularensis*, and CDV [23, 53, 54]. Most investigations of morbilliviruses in polar bears have detected CDV rather than phocine distemper or other marine mammal morbilliviruses; thus, we focused on CDV in this study [43, 55]. Further, in 2019, *T*. *gondii*, *C*. *burnetii*, and *Brucella* spp. were among seven priority zoonotic diseases identified for surveillance in Alaska as part of the U.S. Center for Disease Control One Health Zoonotic Disease Prioritization framework [56].

This dataset allowed us to track changes in pathogen exposure over three decades, coincident with a decline in summer sea ice in the Chukchi Sea and associated increased summer land use by polar bears [37, 57], while comprehensive individual animal data provided important context for evaluating specific risk factors. For a small subset of bears in which data were available (n = 23–37 bears depending on the pathogen), we examined hematological parameters to determine if exposure to individual pathogens was associated with a heightened immune response [34]. Elevation in white blood cell counts often occur in response to infection [58, 59]. Together, these data offer insights into potential changes in the distribution of wildlife and zoonotic pathogens and parasites in a region undergoing rapid environmental change and in a species of conservation concern.

## Methods

Polar bears are challenging to access and sample due to their distribution in remote, arctic sea ice habitats far from coastal areas. We used serum and fecal samples available from polar bears that were captured as part of a suite of ecological studies between mid-March and early May during two periods: “historical” (1987–1994; *n* = 115 adult females) and “contemporary” (2008–2017; *n* = 232 subadult and adult males and females). In the interim period of 1995–2007 samples were not collected from polar bears for a variety of logistical and financial reasons. During 1987–1994, adult females were targeted for sampling and application of collars whereas during 2008–2017 all sex and age classes were sampled. Here we describe how these data were used to address each objective (see also Table 1).

**Table 1:** Analyses conducted to determine if pathogen exposure of Chukchi Sea polar bears changed over time and to identify whether exposure might be associated with differences in diet, summer land use, and/or sex/age. Because adult females were targeted in the historic time period, analyses including historic data were restricted to adult females to avoid potential bias of differences in sex/age class sampling. Cubs were excluded from all analyses due to the potential lack of independence between dependent cubs and mothers. Land use could only be determined for adult females because they are the only sex and age class in which collars can be safely deployed to track habitat use. Separate analyses were conducted because not all data were available for all bears. Samples sizes are provided for specific analyses in tables of results.

Question	Objective	Males or females	Historic or contemporary	Analysis	Dependent variable	Independent variable	Covariates
Changes over time	Determine if pathogen exposure increased over time regardless of diet or age/sex effects	Females	Both	Binary logistic regression	Pathogen exposure (binary)	Period	None
Effects of land use	Determine if pathogen exposure differed for bears that did and did not summer on shore	Females	Both	Binary logistic regression	Pathogen exposure (binary)	Summer land use (binary)	Period
Age/sex effects	Determine if pathogen exposure differed between adults and subadults and males and females	Both	Contemporary	Binary logistic regression	Pathogen exposure	Sex and Age Class	None
Diet effects—isotopes	Determine if dietary differences reflected in hair isotopes are associated with pathogen exposure	Females	Both	Mann-Whitney U test	Hair δ^13^C and δ^15^N	Pathogen exposure	Period
Diet effects—isotopes	Determine if dietary differences reflected in hair isotopes are associated with differences in pathogen exposure	Both	Contemporary	Binary logistic regression	Pathogen exposure	Hair δ^13^C and δ^15^N	None
Diet effects -fatty acids	Determine if differences in diet prey contributions based on quantitative fatty acid analysis are associated with differences in pathogen exposure	Both	Contemporary	Mann Whitney U test	Dietary prey proportion	Pathogen exposure	None

To address Objective 1 evaluating whether pathogen exposure changed in the Chukchi Sea polar bear population between 1987–1994 and 2008–2017, we compared fecal parasites and serum antibody prevalence between adult females sampled during both time periods (Table 1). Less than 10 adult males and subadults were sampled during 1987–1994 and therefore were excluded from the analysis. Mean age of females did not differ between those sampled during the two time periods (one-way ANOVA: F_1,173_ = 0.09, p = 0.77). No covariates were included in this analysis because the objective was to determine if pathogen exposure changed over time regardless of individual factors that might affect pathogen exposure.

For Objective 2, we sought to determine whether individual pathogen exposure was associated with summer land use, diet, and/or sex and age. Summer land use data were available only for a subset of adult females that were collared and tracked during the two time periods (Table 1). Thus, we compared pathogen exposure among this specific set of sampled females. Similarly, diet information from stable isotopes in hair (as described further below) was available in sufficient sample sizes for both time periods only for adult females. Relationships between summer land use, hair isotopes and pathogen exposure were examined with and without a period effect as highlighted below. Bears sampled during 2008–2017 represented all sex and age classes and a fat sample was collected that allowed dietary analysis based on quantitative fatty acid analysis (QFASA; see details below). We examined sex and age class associations with pathogen exposure independent of dietary effects in separate analyses. Finally, we had a small sample of mother-cub pairs in which we examined the potential for antibody transfer and/or associated exposure between females and cubs.

We addressed Objective 3, using available hematology data that were collected only for a subset of contemporary bears.

### Sample collection

Polar bears were sampled using methods previously described [32, 60] and summarized here. Polar bears were immobilized with Telazol^®^ delivered via a Palmer-Cap-Chur dart rifle (Palmer Cap-Chur Equipment, Douglasville, GA, USA). Polar bears were sampled during both time periods from areas that occurred within the range identified for the Chukchi Sea population by the International Union for the Conservation of Nature Polar Bear Specialist group (Fig 1). During 1987–1994, adult female polar bears were targeted for sampling off the coast of Wrangel Island in Russia, near St. Lawrence Island in the Bering Strait, and off the northwest coast of Alaska as part of a study focused on reproductive success and bear movements. Because adult males have necks that are larger than their heads, tracking movements via telemetry on collars was only possible with adult females. From 2008 to 2017, all sex and age classes of polar bears were sampled off the northwest coast of Alaska. Although some bears sampled during 1987–1994 occurred in areas that were not sampled during 2008–2007 (Fig 1), location data from tagged adult females showed that they regularly move throughout all regions that were sampled [31, 61], including terrestrial habitats in Alaska and Russia [32], such that pathogen exposure would likely not be affected by sampling location. To confirm this, we compared pathogen exposure between the three areas where females were sampled during 1987–1994. There was no difference in exposure for any pathogens between areas sampled (binary logistic regression with region as a factor: p > 0.3 for all analyses; n = 87). Therefore, in comparing pathogen exposure between time periods we did not include a regional effect in the analysis because not all three regions were sampled during both time periods. Studies were conducted under U.S. Fish and Wildlife Service research permits MA 690038 and 046081 and followed protocols approved by the Animal Care and Use Committee of the U.S. Fish and Wildlife Service. Methods for all aspects of sample collection from polar bears were carried out in accordance with relevant guidelines and regulations and approved as specified in the above sections relating to sampling of wild polar bears. In addition, all aspects of the study are reported here in accordance with ARRIVE guidelines (https://arriveguidelines.org).

Upon first capture a vestigial premolar was extracted to estimate age by counting cementum annuli [62], except for cubs which were aged based on body size and dentition. Bears were aged to the nearest year (i.e., integer values). When reporting age differences in pathogen exposure we classified bears ≥5 years old as adults and bears 3–4 years old as subadults. Blood was collected from femoral or jugular vasculature for all bears in no-additive tubes and tubes containing ethylenediaminetetraacetic acid (EDTA; anticoagulant). Blood collected in no-additive tubes was centrifuged the same day of collection to separate blood serum from red blood cells. Adipose tissue and fecal samples were collected from bears sampled 2008–2017. Adipose tissue samples were collected approximately 15 cm lateral to the base of the tail using a 6-mm biopsy punch. Fecal samples were collected either from defecations during or just prior to immobilization or via direct sampling from the rectum using a latex glove.

### Serology and parasite detection

#### Serology

We tested for *C*. *burnetii*, *Leptospira* spp., *N*. *caninum*, and CDV at the Colorado State University Veterinary Diagnostic Laboratory using previously established methods (S1 File). Similarly, testing for antibodies to *B*. *abortus/suis* and *B*. *canis*, was conducted at the Colorado Department of Agriculture Rocky Mountain Regional Animal Health Laboratory using validated methods (S1 File). Antibodies to *T*. *gondii* were evaluated using a previously validated MAT at the Animal Parasitic Diseases Laboratory, Beltsville Agricultural Research Center (Beltsville, MD, USA) with a cutoff titer of 1:25 [63]. This cut-off was previously used to identify *T*. *gondii* antibodies and exposure in polar bears [23] and brown bears (*Ursus arctos*) [53]. We screened sera for *F*. *tularensis* antibodies at the U.S. Geological Survey (USGS) Alaska Science Center using a commercially available febrile antigen agglutination test per the manufacturer’s recommended protocol (Becton, Dickinson and Company, Sparks, MS, USA) and as previously described [23, 64](see S1 File for additional details).

Archived samples are a valuable resource for evaluating potential changes in animal condition, diet, or pathogen exposure over time. However, it is important to account for potential artifacts that may result from differences in storage conditions or analytical methods. Samples from this study were stored at -80°C freezer and kept frozen until analysis, thereby minimizing the potential for degradation. Further, antibody prevalence in sera has been documented to be unaffected by long-term storage for some pathogens, including *T*. *gondii* [65, 66]. To assure consistency in analytical methods, all samples from both temporal periods were analyzed at the same time, in the same laboratories, and with identical methods. Additionally, prior serosurveys conducted on Chukchi Sea polar bears corroborate our historical results and suggest that antibody detection has not been significantly compromised over time [38, 67].

#### Parasitology

We analyzed fecal samples for *Giardia* and *Cryptosporidium* by immunofluorescence assay at the Colorado State University Veterinary Diagnostic Laboratory using previously established methods [68]. We considered a sample to be positive if any *Giardia* cysts or *Cryptosporidium* oocysts were detected (S2 File). In addition to screening fecal samples for the presence of *Giardia* cysts or *Cryptosporidium* oocysts, we screened for other intestinal parasites in a small subset of samples that had adequate volume (*n* = 9) using a modification of [69] double sugar centrifugation technique (additional details in S2 File).

### Seroprevalence over time

We compared pathogen exposure among adult females sampled during historical and contemporary periods using a binary logistic regression with pathogen exposure as the dependent, binary variable and time period as a factor. We report counts as potentially differing when p < 0.1 but report p-values and effect sizes for all results [70]. Although p-values of 0.05 have conventionally been used to denote significance, this cut-off value is arbitrary and recent studies have supported considering more flexible cut-offs in p-values relative to power and sample sizes [70–72]. Because our study involved comparing proportions (i.e., binomial data) with relatively low sample sizes, our power to detect differences was lower and warranted considering differences at p values between 0.05–0.1 as indicative of potential differences [72]. Other covariates, such as dietary indicators and summer land use, were not included in this analysis because we sought only to determine if pathogen exposure changed between the two time periods.

### Factors affecting individual serostatus

#### Summer land use

We determined land use for 118 adult females (sample sizes for associations with individual pathogens varied) that were fit with Argos or GPS satellite-tracking collars from both the historic and contemporary periods [31, 32]. Location data were used to identify adult females that selected land as a summer habitat for ≥21days between July and October when sea ice is at its annual minimum [31, 32] (Additional details in S3 File).

We compared seroprevalence between females that summered on land versus those that summered on the sea ice using a binary logistic regression with summer habitat as a binary factor and with and without period as an additional factor to control for potential differences in exposure between periods.

#### Age and sex

We compared differences in the frequency of pathogen exposure between adult and subadult male and female polar bears and relative to age using a binary logistic regression with pathogen exposure as the dependent, binary variable, sex as a factor, and age as a continuous covariate. For this analysis, only contemporary samples were included because all sex and age classes were targeted for sampling during that period. We included a sex by age interaction if the interaction was significant at p ≤ 0.1. We excluded cubs from analyses because their pathogen exposure could be influenced by maternal behavior or direct transmission but we describe pathogen exposure for mother-cub pairs within the data set. For bears that were sampled on more than one occasion (*n* ≤ 15 depending on the pathogen), we used data from the most recent sampling event for analysis. When discussing age effects, we summarized results for adults (≥5 years of age) and subadults (independent bears age 2–4 years).

#### Diet

We examined hair isotopes (δ^15^N and δ^13^C) of adult females sampled during the two time periods as an indicator of potential dietary differences associated with pathogen exposure. δ^13^C in polar bear hair varies in response to the proportion of blubber versus muscle in the diet as well as differences among marine prey [73]. δ^13^C and δ^15^N are higher in ringed seal tissues than bearded seal, walrus, and whales, and δ^15^N is lower in walrus and bowhead whales compared to both seal species [73]. δ^15^N values of the marine mammal prey (12–18‰) [73] polar bears hunt on the sea ice are approximately twice that of terrestrial-based prey (3–7‰) [74] (see additional details in S4 File). Polar bears in the Chukchi Sea primarily rest when summering onshore and spend little time feeding [75] consistent with studies in other parts of their range where terrestrial foods contribute minimally to meeting energetic needs [76]. We compared δ^13^C and δ^15^N measures in hair between seropositive and seronegative adult female polar bears from both time periods using a Mann-Whitney U-test because variances were not homogenous. In addition, we examined whether serum δ^15^N and δ^13^C affected seropositivity of bears of both sexes sampled 2008–2017 using a binary logistic regression to determine if diet plays a role in pathogen exposure while eliminating potential period differences in pathogen exposure. Prey dietary contributions were not estimated via isotopic modeling because prey samples were not available from the historical period to verify isotopic composition.

We previously determined diets of individual bears sampled from 2008–2017 using quantitative fatty acid analysis (QFASA) which estimates dietary prey contributions from the fatty acid signatures in adipose tissue samples of polar bears and blubber samples of their prey [77]. Because fatty acids in polar bears turn over approximately every 100 days [78], adipose tissue samples collected from bears captured mid-March through May represented diet from approximately December through the date of capture. Prey species included in diet models were determined based on known species overlap and observation data as previously described [77]. For each pathogen of interest, contributions of prey species from QFASA were compared between seropositive bears and seronegative bears using a Mann-Whitney U-test. We attempted a Hellinger transformation (square root of each dietary proportion) [79] to reduce skewness, but data were not normally distributed even after transformation, prompting the use of a non-parametric test.

### Immune response

Hematology data, including cell counts for white blood cells, neutrophils, lymphocytes, monocytes, and eosinophils, were available for a subset of bears sampled 2009–2011, including five dependent cubs (n = 23–37 depending on pathogen). Within 12 hours of capture, whole blood samples collected in EDTA were analyzed using a veterinary hematology analyzer (HM5, Abaxis, Union City, CA, USA). For each pathogen, we compared cell counts between seropositive and seronegative bears using either an ANOVA or Mann-Whitney U-test depending on whether data were normally distributed and had homogenous variances.

For all analyses, normality was tested with a Shapiro-Wilks test and homogeneity of variance was tested with a Levene’s test to determine when non-parametric tests were required. For regression analyses, we examined residuals to ensure linearity and absence of outliers. Analyses were conducted in SPSS (Version 28.0.1.0, IBM).

## Results

### Antibody seroprevalence and fecal parasite detection

**Fig 2:**
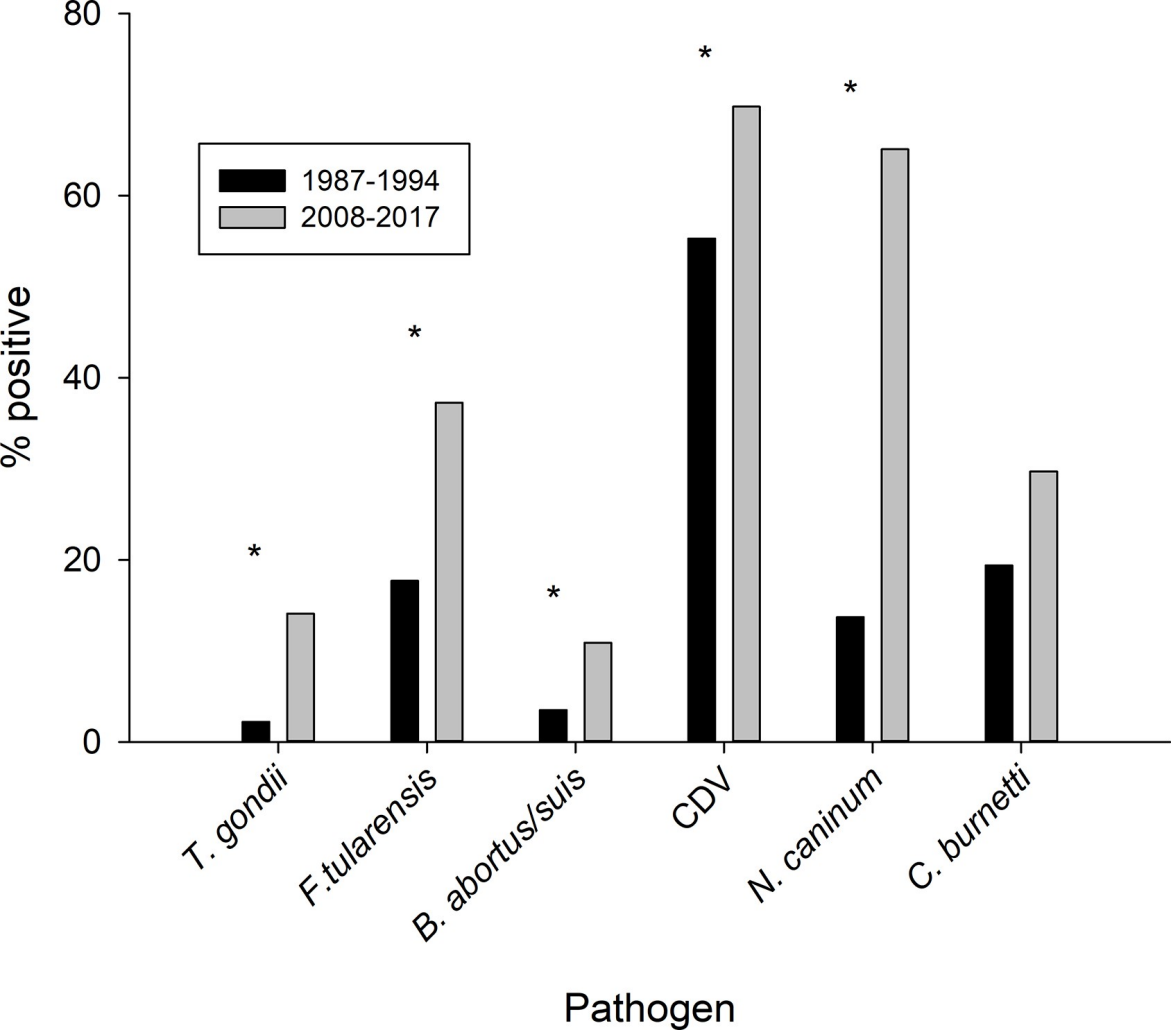
Comparison of the percent of adult and subadult Chukchi Sea polar bears identified as exposed to six pathogens between 1987–1994 and 2008–2017. Asterisks identify differences that were significant (p < 0.1) in a binary logistic regression (CDV = canine distemper virus).

We detected serum antibodies for *T*. *gondii*, *N*. *caninum*, *F*. *tularensis*, *C*. *burnetii*, *B*. *abortus/suis*, and canine distemper virus (CDV) in both historical and contemporary samples (Fig 2, Table 2). Seroprevalence was highest for CDV and lowest for *B*. *abortus/suis* (Table 2). We did not detect *B*. *canis* (*n =* 239) or *Leptospira* sp. (*n =* 244) in serum samples from either period or *Giardia* spp. or *Cryptosporidium* spp. in fecal samples (*n =* 49). No other parasites were detected by fecal float (*n* = 9).

**Table 2:** Results comparing seroprevalence of antibodies (representing % positive) to six pathogens among adult female polar bears sampled during 1987–1994 and 2008–2017 in the Chukchi Sea using a binary logistic regression. Intercepts (*β*) are provided ± standard error.

Pathogen	n	Seroprevalence % (n)		% change	Period effect		
		1987–1994	2008–2017		*Χ* ^*2*^	*p*	*β* ± SE
*Toxoplasma gondii*	157	2.2 (93)	14.1 (64)	540.9	6.30	0.01	2.01 ± 0.80
*Francisella tularensis*	147	17.7 (96)	37.3 (51)	110.7	6.63	0.01	1.02 ± 0.39
*Brucella abortus/suis*	151	3.5 (87)	10.9 (64)	211.4	3.02	0.08	1.24 ± 0.71
Canine distemper virus	139	55.3 (76)	69.8 (63)	26.2	3.07	0.08	0.63 ± 0.36
*Neospora caninum*	158	13.7 (95)	65.1 (63)	375.2	38.20	<0.01	2.46 ± 0.40
*Coxiella burnetii*	157	19.4 (93)	29.7 (64)	53.1	2.22	0.14	0.57 ± 0.38

There were up to 15 pairs of samples analyzed for a given pathogen from the same individuals such that consistency of seroprevalence could be examined between 1–4 years between sampling. Five bears that were seropositive for *T*. *gondii* at initial capture, were positive when recaptured including two females that were each captured three times over four years. Similarly, antibodies to CDV persisted for up to 4 years among eight bears that initially tested positive. In contrast, some bears that were initially positive for *N*. *caninum*, *C*. *burnetii*, and *B*. *abortus/suis* tested negative for antibodies when recaptured one to four years later (S1 Table).

### Seroprevalence over time

Seroprevalence was 26–541% higher in contemporary samples as compared to historical samples for five out of six pathogens detected (Fig 2, Table 2). The seroprevalence of *T*. *gondii*, *F*. *tularensis*, and *N*. *caninum* was significantly higher in adult females sampled 2008–2017 compared to those sampled 1987–1994 (*p* ≤ 0.01; Fig 1; Table 2). The seroprevalence of *B*. *abortus/suis* and CDV was also higher in the contemporary versus historical period (*p* = 0.08 for both comparisons; Table 2). There was no difference in the prevalence of *C*. *burnetii* between time periods (Fig 2, Table 2).

### Factors affecting antibody seroprevalance

#### Summer land use

There were no differences in seroprevalence for any pathogens examined between adult females that summered on land versus those that summered on the sea ice (Table 3); this pattern held both with and without a period effect. Results in Table 3 are presented from models excluding the period effect.

**Table 3:** Results comparing seroprevalence of antibodies (representing % positive) among adult female polar bears sampled 1987–1994 and 2008–2017 in the Chukchi Sea that summered on land versus those that summered on the sea ice using a binary logistic regression. Sample sizes of females that summered on sea ice or land for each pathogen are provided in parentheses.

Pathogen	n	Seroprevalence (%) (n for habitat)		Summer habitat effect	
		Sea ice	Land	*Χ* ^*2*^	*p*
*Toxoplasma gondii*	116	7.6 (79)	5.4 (37)	0.19	0.67
*Francisella tularensis*	109	17.8 (85)	30.6 (24)	2.24	0.14
*Brucella abortus/suis*	113	6.6 (76)	8.1 (37)	0.09	0.77
Canine distemper virus	104	67.1 (70)	58.8 (34)	0.69	0.41
*Neospora caninum*	118	32.5 (80)	47.4 (38)	2.41	0.12
*Coxiella burnetii*	116	19.2 (78)	26.3 (38)	0.75	0.39

#### Age and sex

The seroprevalence of *T*. *gondii* and CDV increased with age (Table 4). Seroprevalence of *T*. *gondii* was more than twice as high in adults (≥ 5 years of age: 17%; *n =* 145) compared to subadults (independent bears age 2–4 years: 8%; *n =* 39). Similarly, we detected CDV in 75% of adults (n = 139) compared to 41% of subadults (*n =* 37). In contrast, seroprevalence of *C*. *burnetii* decreased with age and was more common in subadults (23%; *n =* 39) compared to adults (14%; *n =* 141). Females had higher seroprevalence than males for *F*. *tularensis* (36.8% and 21.9%, respectively; *n* = 121), *B*. *abortus/suis* (9.1 and 2.9%, respectively; *n* = 181), and *C*. *burnetii* (24.7 and 10.6%, respectively; *n* = 180; Table 4).

**Table 4:** Results from a binary logistic regression examining differences in the seroprevalence of antibodies for six pathogens (representing the % positive) between male and female adult and subadult polar bears (sex as a binary fixed effect) sampled 2008–2017. Age was included as a continuous covariate. Interactive effects between sex and age were included if significant at *p* < 0.1. Intercepts (*β*) are provided ± standard error (SE) only when effects were significant at *p* < 0.1. Positive *β* for age indicate increasing frequency of pathogen exposure with age and positive *β* for sex indicate higher frequency of exposure in females compared to males.

Pathogen	N	Total % positive	Sex					Age		
			Females Seroprevalence (n)	Males Seroprevalence (n)	*Χ* ^*2*^	*p*	*β* ± SE	*Χ* ^*2*^	*p*	*β* ± SE
*Toxoplasma gondii*	184	32.3	32.7 (76)	38.4 (108)	2.2	0.14		19.2	<0.01	0.15 ± 0.03
*Francisella tularensis*	121	28.9	36.8 (57)	21.9 (64)	3.79	0.05	0.81 ± 0.42	0.84	0.36	
*Brucella abortus/suis* *	181	5.5	9.1 (77)	2.9 (104)	5.21	0.02	3.65 ± 1.60	0.10	0.76	
Canine distemper virus	177	67.8	65.8 (76)	69.3 (101)	0.7	0.42		10.2	<0.01	0.10 ± 0.03
*Neospora caninum*	183	64.7	69.7 (76)	61.1 (107)	1.88	0.17		1.87	0.17	
*Coxiella burnetii*	180	16.6	24.7 (74)	10.6 (106)	8.27	<0.01	1.26 ± 0.44	3.94	0.05	-0.08 ± 0.04

*Significant sex-age interaction.

Serologic results were not consistent across mother-cub pairs for any of the six pathogens we detected (S2 Table). *F*. *tularensis*, *B*. *abortus/suis*, *N*. *caninum and C*. *burnetii* antibodies were sometimes present in cubs but not in their mothers, whereas in other cases we detected the opposite pattern (S2 Table).

#### Diet

We did not detect any isotopic differences in the hair of adult females (the only sex and age class in which samples were available for both time periods) between historical and contemporary periods or in association with pathogen exposure (S3 Table). There was also no effect of hair δ^15^N or δ^13^C on pathogen exposure of both males and females sampled during the contemporary period for any of the six pathogens detected (*p* ≥ 0.1 in binary logistic regressions). Hair δ^15^N and δ^13^C values ranged from 19.4 to 22.3‰ (*n* = 168) and -14.9 to -17.1‰ (*n* = 177), respectively, consistent with consumption of marine mammal prey. δ^15^N in hair of adult females did not differ between the two time periods (Mann-Whitney U: 1150.0, *n =* 123, *p* = 0.20) or δ^13^C (*U* = 1674.0, *n =* 126, *p* = 0.25); therefore, isotopic comparisons did not include period as a factor.

In contrast, prey contributions to the diet of subadult and adult males and females sampled 2008–2017 based on previously published diet estimates derived from tracking fatty acids in polar bear tissues relative to their prey [73, see methods for more details on diet analysis] differed for *T*. *gondii*, *N*. *caninum*, *F*. *tularensis*, and *C*. *burnetii*, but not for *B*. *abortus/suis* and CDV. Bears seropositive for *T*. *gondii* (*n =* 153) consumed more bearded seal (33 vs. 22%) and less adult ringed seal (31 vs. 44%) than seronegative bears (*n =* 24; Table 5). Bears that were seropositive for *F*. *tularensis* (*n =* 33) consumed less ringed seal pup (9 vs. 12%) than those that were seronegative (*n =* 81). Consumption of bowhead whale was higher (7.3%; *n* = 113) among bears that were seropositive for *N*. *caninum* compared to those were seronegative (4.5%; *n* = 63). Bears that were seropositive for *C*. *burnetii* (*n =* 28) consumed less bearded seal (14 vs 25%), less beluga whale (9 vs 17%) and more adult ringed seal (61% vs. 39%) than bears that were seronegative (*n =* 145).

**Table 5:** Statistical results from comparing prey percentages in diets of subadult and adult male and female polar bears sampled in the Chukchi Sea 2008–2017 that tested positive or negative for pathogen exposure using Mann-Whitney U-tests. Dietary prey percentages are based on quantitative fatty acid analysis using data from polar bear and prey adipose tissue samples as reported in [79]. Sample sizes for each analysis are provided in parentheses within the pathogen column. P-values in bold with an asterisk highlight significant dietary percentages between bears exposed versus not exposed to a pathogen.

Pathogen	Statistic	Bearded seal	Beluga whale	Bowhead whale	Adult ringed seal	Ringed seal pups
*Toxoplasma gondii* (177)	*Positive*	**33.3**	14.8	9.1	**31.1**	11.7
	*Negative*	**21.8**	16.0	5.8	**44.4**	12.0
	*U*	**2327.0**	1776.0	2106.0	**1330.5**	1812.0
	*p*	0.04*	0.80	0.23	0.03*	0.92
*Francisella tularensis* (114)	*Positive*	24.5	14.3	5.3	46.8	**9.2**
	*Negative*	24.0	15.0	8.1	40.2	**12.8**
	*U*	1413.5	1318.0	1078.5	1501.0	**947.5**
	*p*	0.63	0.91	0.10	0.30	0.02*
*Brucella abortus/suis* (174)	*Positive*	21.3	13.3	5.2	51.2	8.9
	*Negative*	23.7	15.8	6.4	42.1	12.0
	*U*	760.5	664.0	810.0	969.5	591.5
	*p*	0.70	0.31	0.95	0.33	0.14
Canine distemper virus (170)	*Positive*	23.4	15.2	6.6	42.9	11.9
	*Negative*	24.4	16.6	5.8	41.4	11.8
	*U*	3070.5	3127.0	3303.0	3244.5	3149.0
	*p*	0.76	0.91	0.63	0.79	0.96
*Neospora caninum* (176)	*Positive*	23.2	15.2	**7.3**	42.4	11.8
	*Negative*	23.8	17.0	**4.5**	42.5	12.3
	*U*	3596.0	3257.0	**4239.0**	3522.0	3452.0
	*p*	0.91	0.35	0.03*	0.91	0.74
*Coxiella burnetiid* (173)	*Positive*	**13.9**	**9.4**	5.5	**61.2**	10.0
	*Negative*	**25.1**	**17.0**	6.4	**39.2**	12.3
	*U*	**1400.0**	**1354.0**	2044.0	**2918.0**	1723.5
	*p*	<0.01*	<0.01*	0.95	<0.01*	0.21

### Immune response

Because our sample of bears with white blood count data was relatively small (*n =* 38), we report counts as potentially differing when *p* < 0.1 (S4 Table). Monocytes were higher in bears seropositive for CDV (450 vs. 320/μL; *n =* 22) and *N*. *caninum* (440 vs. 320/μL; *n =* 23) but were lower in bears seropositive for *F*. *tularensis* (140 vs. 400/μL; *n =* 3) compared to those that were seronegative. Lymphocytes were higher (207 vs. 142/μL) in bears seropositive for *T*. *gondii* (*n =* 3) whereas total white blood cell counts and neutrophils were higher (WBC = 11.1 vs. 8.6 x 10^3^/ μL; NEU = 8.6 vs. 6.1 x 10^3^/μL) among the few bears that were seropositive for *B*. *abortus/suis* (*n =* 3) compared to those that were seronegative.

## Discussion

**Fig 3:**
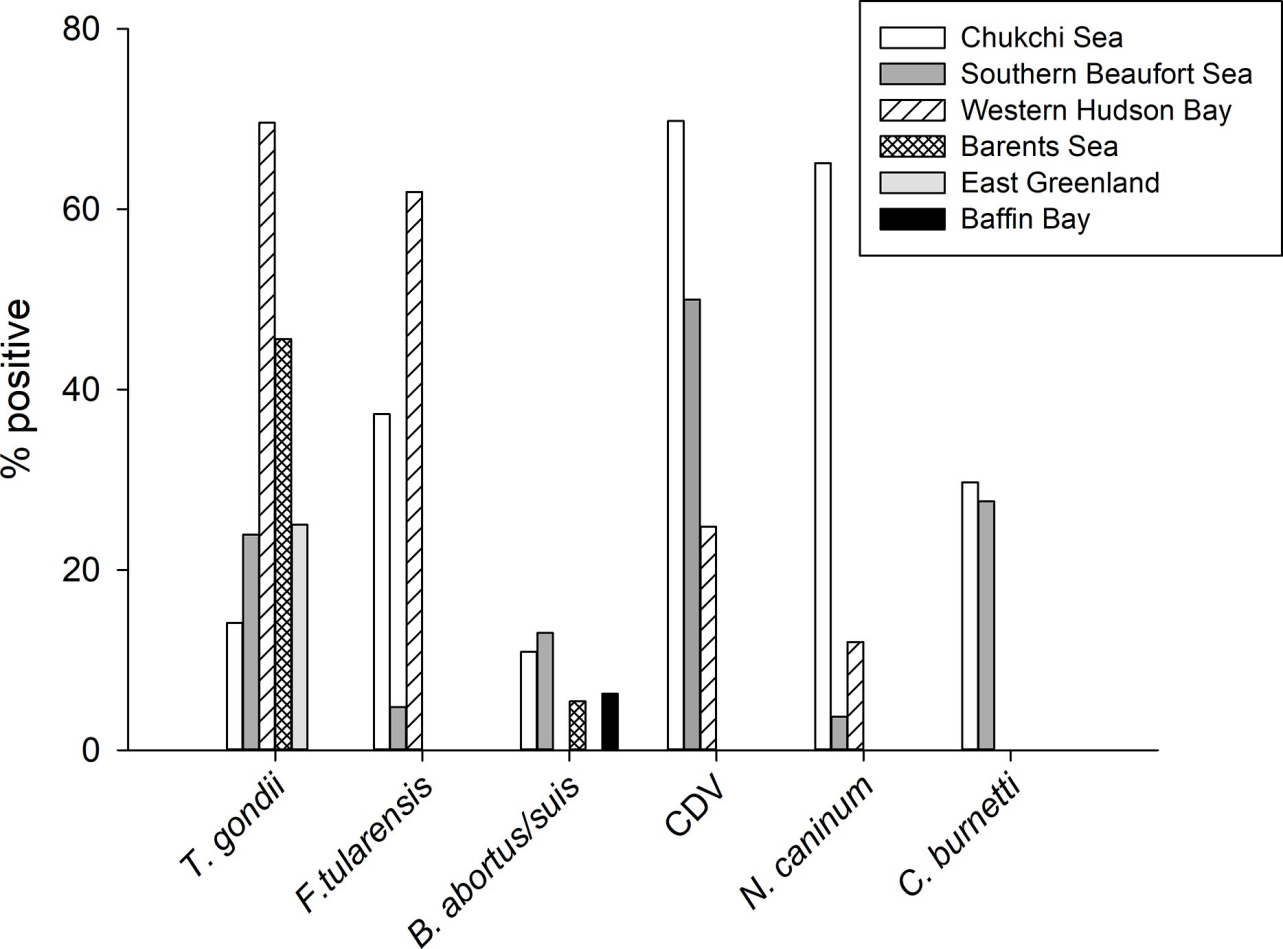
Comparison in seropositivity of six pathogens for contemporary Chukchi Sea polar bears (this study) in comparison to other populations for which data are available (Southern Beaufort Sea) [23, 43, 65, 99]; Western Hudson Bay [24]; Barents Sea [63; 106]; East Greenland [106, 107], and Baffin Bay [106].

We detected antibodies to six pathogens of wildlife and zoonotic concern in Chukchi Sea polar bears and identified increases in seroprevalence for *T*. *gondii*, *N*. *caninum*, *F*. *tularensis*, *B*. *abortus/suis*, and CDV between 1987–1994 and 2008–2017. The magnitude and timing of these increases—ranging from 30% to 541% over a span of two to three decades (Fig 2)—represent some of the most rapid changes in pathogen exposure reported among polar bears to date. Seroprevalence of the protozoan parasites *T*. *gondii* and *N*. *caninum* was several times higher in contemporary versus historical samples, representing more than six- and nearly five-fold increases, respectively. Exposure to the bacterial pathogens *F*. *tularensis* and *B*. *abortus/suis* more than doubled, whereas exposure to viral pathogen CDV increased by about one-third. Seroprevalence of bacterium *C*. *burnetii* appeared to follow a similar trajectory but was not statistically different between the two periods. By contrast, seroprevalence for an overlapping suite of pathogens reported in the Western Hudson Bay polar bear population increased ~30–60% over a similar timeframe (1986 to 2017) [24; Fig 3]. Among the pathogens included in our study, increases in western Hudson Bay bears were noted for *T*. *gondii* and *F*. *tularensis*, but not for *N*. *caninum* or CDV [24]. For Southern Beaufort Sea bears, seroprevalence of *T*. *gondii* and *Brucella* spp. increased by 81% and 27%, respectively, over an 8-year interval [23]. Taken together, these results suggest that polar bears, and potentially other species in Arctic ecosystems, may be facing growing exposure to parasitic, bacterial, and viral pathogens, and that regional differences exist.

*F*. *tularensis*, CDV, and *N*. *caninum*, the three pathogens with the highest overall seroprevalence among contemporary Chukchi Sea bears and moderate to high prevalence compared to other polar bear populations (Fig 3), are all considered to be primarily of terrestrial origin. We detected antibodies to the bacterial pathogen *F*. *tularensis* in more than a third of contemporary polar bear samples despite the relative paucity of reports in marine species [80]. Exposure to CDV, the single viral pathogen included in our study, was high among Chukchi Sea bears, with more than two thirds of the contemporary population seropositive. CDV is thought to be enzootic in some parts of the Arctic occurring in brown bears (*Ursus arctos*) [53] and wild canids, including Arctic foxes [45], which are suspected to play an important role in transmission [81, 82]. Additionally, domestic dogs can be a source of infection, especially in areas where vaccination is limited [82]. Over half of Chukchi Sea bears sampled in 2008–2017 had antibodies to *N*. *caninum*, a rate much higher than that reported for other polar bear populations (Fig 3). Canids are the only definitive hosts but caribou have also tested seropositive [83].

In contrast, seroprevalence of the closely related parasite *T*. *gondii* was lower in contemporary Chukchi Sea bears compared to other polar bear populations sampled over the past two decades (Fig 3). *T*. *gondii* occurs commonly in both marine and terrestrial environments, including in ringed and bearded seals in the adjacent Beaufort Sea [40] and southern Chukchi Sea [50, 51]. Land use has been positively associated with exposure in other polar bear populations [23, 24]. *T*. *gondii* has been detected in Arctic fox in Svalbard and associated with their potential predation on migratory birds, such as geese [84]. Thus, Chukchi Sea polar bears may be exposed both in marine and terrestrial environments.

Relatively few surveillance efforts have targeted *C*. *burnetii* in Arctic wildlife, but seroprevalence among Chukchi bears was comparable to that reported from the Southern Beaufort Sea in 2007–2014 (Fig 3). *C*. *burnetii* is common in ruminants and has been detected in various marine mammals in Alaska, though its full host and geographic range is unknown [53]. The bacterium is expelled during parturition and is highly persistent in the environment, meaning that infected animals need not be present contemporaneously for transmission to occur [85].

Seroprevalence of *B*. *abortus/suis* in our study was slightly lower or similar to other polar bear populations where data are available (Fig 3). Though *Brucella* type is not specified in all reports, data are generally comparable across studies because assays use non-species specific antigens [39]. *Brucella* spp. are commonly detected among marine mammals [39, 86, 87], although the few studies we are aware of that tested for *B*. *canis* in Arctic marine mammals did not find evidence of exposure, including a study of ringed seals in Nunavut [35]. Seroprevalence in Southern Beaufort Sea polar bears was negatively correlated with land use [23]. However, other studies have pointed to terrestrial sources of *Brucella* exposure among polar bears [39, 88] and *Brucella* has increased in some Arctic terrestrial species [39].

We also did not detect *Giardia* or *Cryptosporidium* in fecal samples, or *Leptospira* spp. in sera. To date, the only report of *Giardia* in polar bears was from a single individual in the southern Beaufort Sea [89], while *Cryptosporidium* was reported in a polar bear from an unspecified source [90]. The absence of exposure to *Leptospira* spp. in Chukchi Sea polar bears is somewhat surprising based on relatively high antibody prevalence to *Leptospira* spp. (up to 27.8%) in Chukchi Sea ringed seals [50], although there appears to be little to no exposure of bearded seals [51, 91].

Although exposure to five of six pathogens examined increased over the past three decades in Chukchi Sea polar bears, pathogen exposure was not consistently higher in polar bears in the Chukchi Sea and Western Hudson Bay populations compared to other populations in more northern areas (Fig 3). Chukchi Sea polar bears had the highest exposure to CDV and *N*. *caninum* of any populations measured to date (Fig 3) but because transmission pathways of these two pathogens are thought to be primarily terrestrial, it is unlikely that migration of marine species from southern seas played a role in their high exposure.

Contrary to other studies that identified a link between summer land use and disease exposure among polar bears [23, 24], there were no differences in seroprevalence between adult females that summered on land and those that summered on sea ice for any pathogens in the Chukchi Sea, including pathogens typically considered to be of terrestrial origin. Features of areas where Chukchi Sea polar bears summer on land and their summer land use behavior may contribute to minimizing exposure to terrestrial pathogens. In the Chukchi Sea ≤50% of bears summer onshore [32] and they summer primarily on Wrangel Island in far north Russia [31] where they spend >90% of their time resting [72]. Wrangel Island has a small military base but no other permanent human settlement. In contrast, all bears in Hudson Bay and other parts of eastern Canada where sea ice is seasonal, summer on land. Further, polar bears in both Hudson Bay and the southern Beaufort Sea summer on the mainland in areas with higher human activity and more permanent settlement than other parts of the Arctic which could increase anthropogenic pathways, such as deposition of caribou carcasses from hunting and exposure to domestic species, including canids. The majority of bears in the southern Beaufort Sea visit the remains of subsistence-harvested bowhead whales [14] which concentrate multispecies aggregations, including brown bears, that may contribute to terrestrial pathogen exposure [23]. Thus, although summer land use by Chukchi Sea polar bears has increased, our results suggest it may not be a primary factor explaining the increased pathogen exposure that has occurred over the past 20–30 years, at least among adult females.

Alongside climatic or habitat use trends, individual host factors have been identified as important explanatory variables in models of community disease ecology [92]. Bacterial pathogens *F*. *tularensis*, *C*. *burnetii*, and *B*. *abortus/suis* were more common in females, with seroprevalence of *C*. *burnetii* and *B*. *abortus/suis* more than double that of males. Females have greater exposure to terrestrial-based pathogens because the majority of pregnant females in the Chukchi Sea den on land whereas land use by males is limited to only the fraction of the population that summers onshore [31]. Dietary differences may also contribute to higher prevalence of *C*. *burnetii* in females since they consume less bearded seal and beluga whale and more adult ringed seal [70], all of which were associated with higher seropositivity. In Alaska, *C*. *burnetii* has been detected in ringed seals in the Bering and Chukchi seas [50]. Bears seropositive for the parasite *T*. *gondii* consumed more bearded seal and less adult ringed seal than seronegative bears, consistent with higher prevalence of *T*. *gondii* in older, larger bears that eat more large-bodied bearded seals [27]. Exposure to *N*. *caninum* was positively associated with consumption of bowhead whales, which are primarily available to Chukchi Sea polar bears on land as beached or hunter-harvested carcasses [28, 93].

While diet may affect pathogen exposure, antibody duration plays a role in explaining difference in exposure relative to age. Pathogens that elicit long-lasting antibodies may be more prevalent in older animals because of the longer lifespan over which they are exposed. The frequency of exposure to *T*. *gondii* and CDV were positively correlated with age, with *T*. *gondii* antibodies twice as prevalent in adults as subadults. All five bears that initially tested positive for *T*. *gondii* were positive at recapture including two females that were captured three times. CDV antibodies of 8 bears in our study similarly persisted for up to 4 years. In contrast, seroprevalence of *C*. *burnetii* decreased with age and one of the two bears that initially tested positive was later recaptured and tested negative, consistent with evidence that *C*. *burnetii* antibodies have a relatively short duration, on the order of several months [94].

Although our sample size of mother-cub pairs was small (*n* = 10–17), none of the pathogens we detected were consistently positive across pairs. However, some of the cubs were two-year-olds that may have already been weaned but were still traveling with their mother. Further, transfer of antibodies through milk for at least some of the pathogens has been shown to be dependent on the mother’s phase of infection [95]. CDV and *C*. *burnetii* antibodies were commonly present in both mother and cub, which concurs with documented maternal transfer via lactation [96].

All of the pathogens we detected can cause illness in wildlife [97–101], but tracking specific effects in free-ranging wildlife is challenging. We used hematology data to determine whether the presence of antibodies might be associated with an active immune response. Bears that tested positive to *T*. *gondii*, *N*. *caninum*, *B*. *abortus/suis*, and CDV exhibited elevated white blood cell counts which have been associated with reduced body condition in polar bears [33] and can be an indication of response to inflammation, infection, and stress. Although mean monocyte and lymphocyte values for seropositive and seronegative bears were within ranges observed in healthy American black bears (*Ursus americanus*) [102], lymphocyte levels observed in polar bears seropositive for *T*. *gondii* were nearly twice the levels observed for polar bears in the neighboring southern Beaufort Sea [34]. Neutrophil counts for bears testing positive for *B*. *abortus/suis* were also higher than the mean and standard deviation observed for polar bears in the southern Beaufort Sea [34, 103], It is important to note that sample sizes of seropositive individuals with hematology data for *T*. *gondii*, *B*. *abortus/suis*, and *F*. *tularensis* were small (3–12 bears) and factors besides infection may affect hematology values [103]. Further, measuring serum antibodies, as we did here, detects prior exposure which is not synonymous with active infection.

Prevalence of exposure to five of the six pathogens we examined is relatively similar to the prevalence observed across multiple healthy brown bear populations in Alaska [53], suggesting that, bears may be minimally affected by exposure to many of the pathogens we examined. Further, the Chukchi Sea polar bear population has exhibited stable body condition and recruitment over the past several decades [60, 77] despite the increases in pathogen exposure we observed. Thus, there is no evidence to conclude that pathogen exposure has negatively affected the health of Chukchi Sea bears but increases in seroprevalence alongside apparent immune response highlight the importance of continued surveillance.

This study revealed increases in exposure to wildlife and zoonotic pathogens among a marine apex predator experiencing loss of its sea ice habitat. Given multiple stressors on polar bears in the face of rapid changes to their sea ice habitat, pathogen exposure could serve as a compounding factor if exposure compromises health. Further, four pathogens detected in this study—*T*. *gondii*, *C*. *burnetii*, *B*. *abortus/suis*, and *F*. *tularensis*—are zoonoses that have previously caused human illness in northern communities [8, 9, 19, 104, 105]. The serologic testing we used in this study identified pathogen exposure but did not determine the presence of disease in polar bears or the potential for transmission to humans through subsistence hunting. Surveillance efforts both for monitoring polar bear health and potential for transmission to humans, would be improved by targeted tissue sampling when possible, such as in collaboration with subsistence harvesters [101, 102], to screen for potential signs of disease and transmission via polar bear tissues.

## Supporting information

S1 File Additional serology methods. (DOCX)

S2 File Additional parasitology methods. (DOCX)

S3 File Additional methods using locations to determine summer land use. (DOCX)

S4 File Additional methods measuring isotopes in hair. (DOCX)

S1 Table Serologic results for subadult and adult polar bears in the Chukchi Sea sampled on multiple occasions 1990–2017, showing observed seroconversion (negative to positive) or seroreversion (positive to negative). N is the number of individuals that were positive at any sampling time. Repeated sampling of individuals ranged from 1 to 4 years apart.
(DOCX)

S2 Table Serologic results for six pathogens among mother-cub pairs sampled 1990–1992 and 2008–2017 in the Chukchi Sea. Cubs were either yearlings (1–1.5 years old) or two-year olds (2–2.5 years old). “+” indicates testing positive for antibodies and “-”indicates testing negative for antibodies.
(DOCX)

S3 Table Results from Mann-Whitney U-tests comparing the nitrogen (δ^15^N) and carbon (δ^13^C) isotopes in polar bear hair between adult females sampled 1987–1994 and 2008–2017 that tested positive or negative for exposure to pathogens. Isotopes were used as an indicator of potential dietary differences among bears that tested positive or negative for exposure to each pathogen. Isotopes did not differ between time periods, so time period was not included as a factor in the comparisons. Variances were homogenous but δ^13^C were not normally distributed (Shapiro-Wilks test 0.98, p = 0.04).
(DOCX)

S4 Table Comparisons of total white blood cell (WBC), lymphocyte (LYM), monocyte (MON), neutrophil (NEU), and eosinophil (EOS) counts and the NEU:LYM ratio of 21 adult, 12 subadult and 5 dependent young polar bears sampled in the Chukchi Sea that tested positive or negative for pathogen exposure using ANOVAs or Mann-Whitney U-tests (for data sets that were not homogenous and/or not normally distributed). Sample sizes are provided in parentheses for each pathogen. P-values in bold with asterisks highlighting differences that were significant (p≤0.05) and marginally significant (p<0.10).
(DOCX)
