# Supplementary material for: Increased pathogen exposure of a marine apex predator over three decades
Source: PLoS One. 2024 Oct 23;19(10):e0310973. doi: 10.1371/journal.pone.0310973 (PMC11498681; doi:10.1371/journal.pone.0310973)
Supplement: S1 File — (DOCX) [file pone.0310973.s001.docx]

**Any use of trade, firm, or product names is for descriptive purposes only and does not imply endorsement by the U.S. Government.**

**Supporting Information S1**. **Additional Serology methods**

For detection of *Coxiella burnetii* antibodies, we used a commercially-available enzyme-linked immunosorbent assay (ELISA) kit (IDEXX, Westbrook, ME, USA), which has been shown to correlate well with the USDA complement fixation assay (Emery et al., 2012). The positive result cutoff was set at ≥1:128 and we combined results from *C. burnetii* Phase I and Phase II antibodies. To test for the presence of *Leptospira* spp. antibodies, we performed a leptospirosis microscopic agglutination test (MAT) using antigen from 6 serovars obtained from the National Veterinary Services Laboratories (Ames, IA, USA), with sera diluted at 1:100, 1:200, and 1:400 and added to serovar-specific antigen using 96-well test plates. For *N. caninum*, we used a competitive ELISA (cELISA) for detection of IgG antibodies which has shown high specificity to *N. caninum* and no cross-reactivity with *T. gondii* (Baszler et al., 1996); results are reported as antibodies detected or not detected, which correlates to positive or negative, respectively. For CDV, we used a CDV antibody SN assay with positive titer cutoff at ≥1:16.

Initial screening for *B. abortus/suis* was conducted by buffered acidified plate antigen (BAPA), followed by fluorescence polarization assay (FPA) for samples positive by BAPA. Only detections that were confirmed by FPA with mP≥20 were considered positive. We used the *B. canis* 2-Mercaptoethanol Tube Agglutination Test to screen for antibodies to *B. canis* as described in (CIT TK), with titer values ≥100 considered positive.

We ran serial dilutions of sera ranging from 1:20–1:320 alongside *F. tularensis* antisera to serve as positive controls and scored titers for each dilution based on agglutination present during a visual scan 60 seconds after mixing. Titers ≥1:20 compared to positive controls were considered positive.

**References**

Baszler, T.V., D.P. Knowles, D.P. Dubey, J.M. Gay, B.A. Mathison, and T.F. McElwain. 1996. Serological diagnosis of bovine neosporosis by *Neospora caninum* monoclonal antibody-based competitive inhibition enzyme-linked immunosorbent assay. Journal of Clinical Microbiology 34:1423-1428.

Emery, M.P., E.N. Ostlund, and B.J. Schmitt. 2012, Comparison of Q fever serology methods in cattle, goats and sheep. Journal of Veterinary Diagnostic Investigations 24: 379-382.
