# Supplementary material for: Increased pathogen exposure of a marine apex predator over three decades
Source: PLoS One. 2024 Oct 23;19(10):e0310973. doi: 10.1371/journal.pone.0310973 (PMC11498681; doi:10.1371/journal.pone.0310973)
Supplement: S2 File — (DOCX) [file pone.0310973.s002.docx]

**Supporting Information S2. Additional Parasitology methods**

**Any use of trade, firm, or product names is for descriptive purposes only and does not imply endorsement by the U.S. Government.**

We employed the MERIFLUOR Cryptosporidium/Giardia direct immunofluorescent assay (Meridian Bioscience Inc, Cincinnati, Ohio) per manufacturer's directions, with positive and negative controls for each batch. We examined slides at 100×, and if cysts or oocysts were not detected, then also at 200× magnification using a fluorescence microscope.

In addition to screening fecal samples for the presence of Giardia cysts or Cryptosporidium oocysts, we screened for other fecal parasites in nine available samples (Foreyt 1997). Briefly, one gram of fecal sample was mixed in approximately 12 ml of de-ionized water and centrifuged; the sediment was then resuspended in Sheather’s sugar solution and the tube topped with a coverslip and centrifuged again. The coverslip was removed and the resulting sediment washed in de-ionized water three times before the sediment was microscopically reviewed for all parasitic elements.

**References**

Foreyt, W.J. 1997. Veterinary Parasitology Reference Manual. Iowa State Univ Press, Ames IA.
