# Supplementary material for: Increased pathogen exposure of a marine apex predator over three decades
Source: PLoS One. 2024 Oct 23;19(10):e0310973. doi: 10.1371/journal.pone.0310973 (PMC11498681; doi:10.1371/journal.pone.0310973)
Supplement: S3 File — (DOCX) [file pone.0310973.s003.docx]

**Supporting Information S3**. **Additional methods using locations to determine summer land use**

**Any use of trade, firm, or product names is for descriptive purposes only and does not imply endorsement by the U.S. Government.**

Per previous analyses, bears summering on land were identified as those that spent ≥21 days onshore between July and October (Rode et al., 2015; 2022). The 21-day threshold was used to quantify the percent of bears in the population that selected land as a summer habitat, without including bears that briefly visited land for a shorter period. Because sea ice retreats away from land, summer land habitat and sea ice habitat are separated by open water such that most bears are typically selecting one of these two discretely separated habitats, particularly at the time of the sea ice minimum in September. Raw Argos location data from collars were first filtered using the Douglas Argos Filter algorithm (Douglas et al., 2012) to remove implausible locations (e.g., those that could not be corroborated with a repeated location transmission within 10 km or movement rates ≥10km/hr) and locations during periods when collars had become detached from bears. Each bear’s filtered Argos or GPS tracking data were fit to a continuous time correlated random walk model that estimated locations every 6 hours using the Program R (R Core Team 2021) package ‘crawl’ (Johnson et al., 2008; Johnson & London, 2018). Land use was identified as any crawl-estimated location that occurred within 5 km of the coast (Rode et al., 2015; Atwood et al., 2016a) as depicted by a global high-resolution digital coastline (Wessel & Smith, 1996; GSHHG, 2020). Locations estimated during intervals of >14 days without any empirical tracking locations were excluded. Periods of land use were not interrupted if crawl locations fell >5 km from land but for <24 hours.

**References**

Atwood, T.C., E. Peacock, M.A. McKinney, K. Lillie, R. Wilson, D.C. Douglas, S. Miller, and P. Terletzky. 2016. Rapid environmental change drives increased land use by an Arctic marine predator. PLoS ONE 11:e0155932.

Douglas, D.C., R. Weinzierl, S.C. Davisson, R. Kays, M. Wikelski, & G. Bohrer. 2012. Moderating Argos location errors in animal tracking data. Methods in Ecology and Evolution 3: 999–1007.

GHSSG. Version 2.3.7. Accessed on November 23, 2020. <https://www.soest.hawaii.edu/pwessel/gshhg/>

Johnson, D.S., J.M. London, M.-A. Lea, & J.W. Durban. 2008. Continuous-time correlated random walk model for animal telemetry data. Ecology 89:1208–1215. https://doi.dor/10.1890/07-1032.1

Johnson, D.S., & J.M. London. 2018. crawl: an R package for fitting continuous-time correlated random walk models to animal movement data. Available from Zenodo at <https://doi.org/10.5281/zenodo.596464>

R Core Team. 2021. R: a language and environment for statistical computing. R Foundation for Statistical Computing, Vienna Austria.

Rode, K.D., R.R. Wilson, E.V. Regehr, M. St. Martin, D.C. Douglas, & J. Olson. 2015. Increased land use by Chukchi Sea polar bears in relation to changing sea ice conditions. PLoS ONE 10:e0142213.

Rode, K.D., D.C. Douglas, T.C. Atwood, G.M. Durner, R.R. Wilson, and A.M. Pagano. 2022. Observed and forecasted changes in land use by polar bears in the Beaufort and Chukchi Seas, 1985-2040. Global Ecology and Conservation 40:e02319

Wessel, P., & W. H. F. Smith, A. 1996. Global Self-consistent, Hierarchical, High-resolution shoreline Database, Journal of Geophysical Research 101:8741–8743.
