# Supplementary material for: Increased pathogen exposure of a marine apex predator over three decades
Source: PLoS One. 2024 Oct 23;19(10):e0310973. doi: 10.1371/journal.pone.0310973 (PMC11498681; doi:10.1371/journal.pone.0310973)
Supplement: S4 File — (DOCX) [file pone.0310973.s004.docx]

**Supporting Information S4.** **Additional methods measuring isotopes in hair**

**Any use of trade, firm, or product names is for descriptive purposes only and does not imply endorsement by the U.S. Government.**

Hair growth in polar bears is estimated to primarily occur from June to August with some slower growth starting as early as April and continuing through October; therefore, these samples largely reflect summer diet (see review in methods of Rode et al., 2022). Hairs were prepared for isotopic analysis by cleaning with 2:1 chloroform and methanol solution and air drying overnight. Dried hair was loaded into 4 x 6 mm silver capsules (Costech Analytical Technologies, Inc.). Elemental and isotopic composition were measured via conventional continuous flow isotope ratio mass spectrometry. Further details on normalization and data quality and control are provided in Stricker et al. (2022).

**References**

Rode, K.D., B.D. Taras, C.A. Stricker, T.C. Atwood, N.P. Boucher, G.M. Durner, A.E. Derocher, et al. 2022. Diet energy density from isotopes in predator hair associated with survival, habitat, and population dynamics. Ecological Applications: e2751. <https://doi.org/10.1002/eap.2751>

Stricker, C.A., K.D. Rode, B.D. Taras, J.F. Bromaghin, L. Horstmann, and L. Quakenbush. 2022. Summer/fall diet and macronutrient assimilation in an Arctic predator. Oecologia 198: 917-931. <https://doi.org/10.1007/s00442-022-05155-2>
