# Supplementary material for: Increased pathogen exposure of a marine apex predator over three decades
Source: PLoS One. 2024 Oct 23;19(10):e0310973. doi: 10.1371/journal.pone.0310973 (PMC11498681; doi:10.1371/journal.pone.0310973)
Supplement: S1 Table — N is the number of individuals that were positive at any sampling time. Repeated sampling of individuals ranged from 1 to 4 years apart. (DOCX) [file pone.0310973.s005.docx]

**S1 Table. Serologic results for subadult and adult polar bears in the Chukchi Sea sampled on multiple occasions 1990–2017, showing observed seroconversion (negative to positive) or seroreversion (positive to negative).** N is the number of individuals that were positive at any sampling time. Repeated sampling of individuals ranged from 1 to 4 years apart.

|  | n | Seroconversion | Seroreversion |
| --- | --- | --- | --- |
| *Toxoplasma gondii* | 5 | 1 | 0 |
| *Francisella tularensis* | 3 | 1 | 0 |
| *Brucella abortus/suis* | 3 | 2 | 1 |
| Canine distemper virus | 9 | 1 | 0 |
| *Neospora caninum* | 15 | 5 | 5 |
| *Coxiella burnetii* | 2 | 1 | 1 |
