# Supplementary material for: Increased pathogen exposure of a marine apex predator over three decades
Source: PLoS One. 2024 Oct 23;19(10):e0310973. doi: 10.1371/journal.pone.0310973 (PMC11498681; doi:10.1371/journal.pone.0310973)
Supplement: S2 Table — Cubs were either yearlings (1–1.5 years old) or two-year olds (2–2.5 years old). “+” indicates testing positive for antibodies and “-”indicates testing negative for antibodies. (DOCX) [file pone.0310973.s006.docx]

**S2 Table. Serologic results for six pathogens among mother-cub pairs sampled 1990–1992 and 2008–2017 in the Chukchi Sea.** Cubs were either yearlings (1–1.5 years old) or two-year olds (2–2.5 years old). “+” indicates testing positive for antibodies and “-“ indicates testing negative for antibodies.

| Pathogen | n | n positive moms | Mother + cub - | Cub + mother - | Both + | Mother +, one cub +, one - |
| --- | --- | --- | --- | --- | --- | --- |
| *Toxoplasma gondii* | 17 | 1 | 1 | 0 | 0 | 0 |
| *Francisella tularensis* | 12 | 6 | 4 | 1 | 2 | 1 |
| *Brucella abortus/suis* | 15 | 2 | 2 | 4 | 0 | 0 |
| Canine distemper virus | 10 | 9 | 4 | 0 | 5 | 0 |
| *Neospora caninum* | 15 | 3 | 3 | 9 | 0 | 0 |
| *Coxiella burnetii* | 16 | 4 | 1 | 3 | 3 | 0 |
