# Supplementary material for: Increased pathogen exposure of a marine apex predator over three decades
Source: PLoS One. 2024 Oct 23;19(10):e0310973. doi: 10.1371/journal.pone.0310973 (PMC11498681; doi:10.1371/journal.pone.0310973)
Supplement: S3 Table — Isotopes were used as an indicator of potential dietary differences among bears that tested positive or negative for exposure to each pathogen. Isotopes did not differ between time periods, so time period was not included as a factor in the comparisons. Variances were homogenous but δ13C were not normally distributed (Shapiro-Wilks test 0.98, p = 0.04). (DOCX) [file pone.0310973.s007.docx]

**S3 Table.** **Results from Mann-Whitney U-tests comparing the nitrogen (δ^15^N) and carbon (δ^13^C) isotopes in polar bear hair between adult females sampled 1987–1994 and 2008–2017 that tested positive or negative for exposure to pathogens.** Isotopes were used as an indicator of potential dietary differences among bears that tested positive or negative for exposure to each pathogen. Isotopes did not differ between time periods, so time period was not included as a factor in the comparisons. Variances were homogenous but δ^13^C were not normally distributed (Shapiro-Wilks test 0.98, p = 0.04).

| **δ^15^N** | **N (total)** | **n (positive)** | **U** | **p** |
| --- | --- | --- | --- | --- |
| *Toxoplasma gondii* | 119 | 2 | 95.0 | 0.67 |
| *Francisella tularensis* | 117 | 24 | 1286.0 | 0.25 |
| *Brucella abortus/suis* | 113 | 5 | 277.0 | 0.92 |
| Canine distemper virus | 102 | 62 | 1371.5 | 0.37 |
| *Neospora caninum* | 120 | 26 | 1065.0 | 0.32 |
| *Coxiella burnetii* | 119 | 24 | 1313.0 | 0.25 |
| **δ^13^C** | **N (total)** | **n (positive)** | **U** | **p** |
| *Toxoplasma gondii* | 118 | 4 | 215.5 | 0.77 |
| *Francisella tularensis* | 119 | 24 | 1050.0 | 0.55 |
| *Brucella abortus/suis* | 116 | 5 | 377.5 | 0.17 |
| Canine distemper virus | 105 | 65 | 1220.5 | 0.60 |
| *Neospora caninum* | 123 | 27 | 1131.5 | 0.31 |
| *Coxiella burnetii* | 122 | 25 | 1218.5 | 0.97 |
