# Supplementary material for: Increased pathogen exposure of a marine apex predator over three decades
Source: PLoS One. 2024 Oct 23;19(10):e0310973. doi: 10.1371/journal.pone.0310973 (PMC11498681; doi:10.1371/journal.pone.0310973)
Supplement: S4 Table — Sample sizes are provided in parentheses for each pathogen. P-values in bold with asterisks highlighting differences that were significant (p≤0.05) and marginally significant (p<0.10). (DOCX) [file pone.0310973.s008.docx]

**S4 Table. Comparisons of total white blood cell (WBC), lymphocyte (LYM), monocyte (MON), neutrophil (NEU), and eosinophil (EOS) counts and the NEU:LYM ratio of 21 adult, 12 subadult and 5 dependent young polar bears sampled in the Chukchi Sea that tested positive or negative for pathogen exposure using ANOVAs or Mann-Whitney U-tests (for data sets that were not homogenous and/or not normally distributed)**. Sample sizes are provided in parentheses for each pathogen. P-values in bold with asterisks highlighting differences that were significant (p≤0.05) and marginally significant (p<0.10).

| **Pathogen** | **Statistic** | **WBC** | **LYM** | **MON** | **NEU** | **EOS** | **NEU/LYM** |
| --- | --- | --- | --- | --- | --- | --- | --- |
| *Toxoplasma gondii*  (36) | *Positive* | 7.9 | **2.1** | 0.3 | 4.9 | 0.3 |  |
|  | *Negative* | 8.6 | **1.4** | 0.4 | 6.3 | 0.5 |  |
|  | *F or U* | 0.20 | **3.06** | 0.60 | 1.11 | 1.64 | 2.93 |
|  | *p* | 0.66 | **0.09*** | 0.44 | 1.64 | 0.21 | 0.10 |
|  |  |  |  |  |  |  |  |
| *Francisella tularensis*  (23) | *Positive* | 6.7 | 1.5 | **0.1** | 4.5 | 0.4 |  |
|  | *Negative* | 8.7 | 1.4 | **0.4** | 6.4 | 0.3 |  |
|  | *F or U* | 0.96 | 0.06 | **5.38** | 1.26 | 0.13 | 1.50 |
|  | *p* | 0.34 | 0.80 | **0.03*** | 0.27 | 0.72 | 0.23 |
|  |  |  |  |  |  |  |  |
| *Brucella abortus/suis*  (35) | *Positive* | **11.1** | 1.6 | 0.5 | **8.6** | 0.3 |  |
|  | *Negative* | **8.3** | 1.5 | 0.4 | **6.1** | 0.3 |  |
|  | *F or U* | **3.31** | 0.07 | 0.31 | **3.54** | 59.0 | 0.87 |
|  | *p* | **0.08*** | 0.80 | 0.58 | **0.07*** | 0.56 | 0.36 |
|  |  |  |  |  |  |  |  |
| Canine distemper virus  (34) | *Positive* | 9.0 | 1.5 | **0.4** | 6.6 | 0.3 |  |
|  | *Negative* | 7.8 | 1.4 | **0.3** | 5.6 | 0.4 |  |
|  | *F or U* | 1.66 | 0.33 | **4.04** | 1.38 | 0.32 | 0.00 |
|  | *p* | 0.21 | 0.57 | **0.05*** | 0.25 | 0.58 | 0.97 |
|  |  |  |  |  |  |  |  |
| *Neospora caninum*  (36) | *Positive* | 8.9 | 1.4 | **0.4** | 6.6 | 0.3 |  |
|  | *Negative* | 8.0 | 1.5 | **0.3** | 5.6 | 0.4 |  |
|  | *F or U* | 1.02 | 0.09 | **3.93** | 1.54 | 2.91 | 0.07 |
|  | *p* | 0.32 | 0.77 | **0.06*** | 0.22 | 0.10 | 0.80 |
|  |  |  |  |  |  |  |  |
| *Coxiella burnetii*  (37) | *Positive* | 8.1 | 1.3 | 0.4 | 6.1 | 0.2 |  |
|  | *Negative* | 8.6 | 1.5 | 0.4 | 6.2 | 0.4 |  |
|  | *F or U* | 0.12 | 0.23 | 0.24 | 0.01 | 62.0 | 0.60 |
|  | *p* | 0.73 | 0.64 | 0.63 | 0.91 | 0.45 | 0.44 |
